# Statistical Methods in Recent HIV Noninferiority Trials: Reanalysis of 11 Trials

**DOI:** 10.1371/journal.pone.0022871

**Published:** 2011-09-07

**Authors:** Philippe Flandre

**Affiliations:** INSERM UMR-S 943, University Pierre and Marie Curie (UPMC) Paris VI and Department of Virology, AP-HP, Pitié-Salpêtrière Hospital, Paris, France; Lerner Research Institute, Cleveland Clinic, United States of America

## Abstract

**Background:**

In recent years the “noninferiority” trial has emerged as the new standard design for HIV drug development among antiretroviral patients often with a primary endpoint based on the difference in success rates between the two treatment groups. Different statistical methods have been introduced to provide confidence intervals for that difference. The main objective is to investigate whether the choice of the statistical method changes the conclusion of the trials.

**Methods:**

We presented 11 trials published in 2010 using a difference in proportions as the primary endpoint. In these trials, 5 different statistical methods have been used to estimate such confidence intervals. The five methods are described and applied to data from the 11 trials. The noninferiority of the new treatment is not demonstrated if the prespecified noninferiority margin it includes in the confidence interval of the treatment difference.

**Results:**

Results indicated that confidence intervals can be quite different according to the method used. In many situations, however, conclusions of the trials are not altered because point estimates of the treatment difference were too far from the prespecified noninferiority margins. Nevertheless, in few trials the use of different statistical methods led to different conclusions. In particular the use of “exact” methods can be very confusing.

**Conclusion:**

Statistical methods used to estimate confidence intervals in noninferiority trials have a strong impact on the conclusion of such trials.

## Introduction

The efficacy of antiretroviral therapy for treatment of HIV-1 infection has improved steadily since the advent of potent combination therapy in 1996 [1]. Introduction of drugs that offer new mechanisms of action with improved safety profiles and lower pill counts has led to highly potent combination, the so-called Highly Active AntiRetroviral Therapy (HAART) [1], [2]. Most recent HIV clinical trials reflect such an improvement since, in naïve patients, rates of HIV RNA below 50 copies/mL over 80–85% have been reported [3], [4], [5] and promising results were found in treatment-experienced patients [6].

Most of the earlier developments have been made by designing and analysing superiority trials. However, high levels of efficacy and inherent difficulty in the use of combinations of triple-drug makes difficult new improvement. Studies of treatment naïve patients indicate that addition of a fourth drug may provide only small incremental benefits [7]. Moreover, failure of the primary endpoint, usually HIV RNA suppression below 50 copies/mL at week 48, is often due to ‘therapeutic failure’ or lost to follow-up rather than genuine virologic failure. Indeed, use of the so-called TLOVR (time to loss of virologic response) implied that patients who prematurely discontinued the study or modified their study treatment before week 48 are considered as failures [8]. Consequently, in most of the HIV clinical trial using the TLOVR algorithm one observed at least 5–10% of ‘non-virologic’ failures.

HIV noninferiority trial has emerged as the new standard design for HIV drug development among antiretroviral-naïve individuals [9], [10] but also in treatment experienced patients [11], [12], [13]. These trials aim to show that a new treatment (new combination) is not worse than the current standard by more than a prespecified margin, the so-called noninferiority margin. Design and interpretation of these trials have been already discussed and criticized in the HIV area [14]. In the analysis and interpretation of studies of non-inferiority at least five factors must be carefully considered to ensure the validity of the study: selection of non-inferiority margin, number of patients needed for the study, control of study sensitivity, definition of population analysis and ethical justification.

In this work, we present some recent HIV noninferiority trials designed for naïve and treatment experienced patients. Results, hypotheses and the use of the different sets of patients are discussed. The different statistical methods used in these trials are briefly described. A reanalysis of these data with the different methods is presented and discussed. The main objective is to investigate whether the choice of the statistical method influence the conclusion of the trials. The choice of the ‘best’ method is discussed in the last section.

## Materials and Methods

### Noninferiority trials

The objective of this work is to investigate the impact of the statistical analysis currently used on results of recent HIV trials. Criteria to select the HIV noninferiority studies were the following: results published or presented in 2010, inclusion of HIV-infected adult patients (>18 years), use of a primary endpoint based on a difference in proportions reflecting efficacy, and not use of a stratified analysis. In trials using a difference in proportions as primary endpoint, the proportion of response (number of patients with response out of the total number of patients) is provided in each arm. Such information is sufficient to compute the difference in proportions, confidence intervals and tests with any statistical method. It is then easy to recover sufficient information to reanalyze data with another method that the one used in the original publication.

### Population sets and analysis

For superiority trials, the full analysis set - intention-to-treat (ITT) population - is recommended because it tends to avoid over-optimistic estimates of efficacy resulting from a per protocol (PP) analysis, since non-compliers included in the full analysis set will generally diminish the estimated treatment effect [15], [16]. Thus, it is often said that the ITT analysis tends to dilute the treatment difference [17] even though not always [18]. If a dilution of the treatment effect is observed, in noninferiority trials, the ITT analysis will increase the risk of falsely claiming noninferiority [15]. Poor adherence, imprecise measurements and processes increase the variability and mask the differences between treatments increasing again the likelihood of falsely accepting non-inferiority. In a noninferiority trial we considered that ITT and PP analyses have equal importance and their use should lead to similar conclusions for a robust interpretation [15], [16]. A reason, however, to consider the ITT analysis as the primary analysis is that the sample size is computed for the ITT analysis since it seems impossible to estimate how many patients will be excluded from the PP population. Nevertheless one difficulty is the wide range of distinct PP analysis or non-ITT analyses.

### Statistical methods

Although a modified hypothesis testing framework exists, reporting of the noninferiority trials is often preferred using the confidence interval approach. Most methods, however, provide equivalently a test statistic and a corresponding confidence interval of the observed treatment difference. Let π_1_ and π_2_ represent the true proportions of patients in success in patients receiving the new treatment and the reference treatment (control group). We are interested in the difference, π_1_−π_2_ = Δ. Null hypothesis for the noninferiority test is H_0_: Δ≤Δ_L_ versus the alternative hypothesis H_1_: Δ>Δ_L_ where Δ_L_ is the pre-specified noninferiority margin [19], [20]. Estimates of π_1_ and π_2_ are noted p_1_ and p_2_ that correspond to the observed proportions of success in the new treatment and control groups, respectively, with δ = p_1_−p_2_, The general framework for the test statistic z is based on z = (δ+Δ_L_)/se(δ) where se(δ) is the standard error of the observed difference. The most simple and popular method, hereafter called the Wald method, is to estimate se(δ) by (p_1_ (1- p_1_)/n_1_+p_2_ (1- p_2_)/n_2_)^1/2^ using the normal approximation [19]. The corresponding confidence interval of the observed treatment difference is given by δ±Z_α/2_ se(δ) where Z_α/2_ is the upper (α/2)^th^ quantile of the standard normal distribution. In this method there is a complete concordance, for both a given noninferiority margin and type I error, in the conclusion based on the lower limit of the δ's confidence interval or on the p value provided by the z statistic.

Four other methods, however, were applied in the analyses of those recent HIV noninferiority trials; Farrington and Manning (FM), Exact, Newcombe, and Miettinen and Nurminen (MN) methods [21], [22], [23], [24]. The FM approach is based on the statistic z described above but with a different estimate of the standard error. As pointed out by Farrington and Manning, the MN statistic is identical to FM except for a term (N-1)/N which is negligible in large samples [22]. The Newcombe method is based on the Wilson score method for the single proportion, without continuity correction [24]. The term ‘exact’ should be used with cautious since different methods have been proposed to compute ‘exact’ confidence intervals for a difference of proportions. The Exact approach used in the PROGRESS study was proposed by Chan and Zhang (CZ) [18], [21] and provides exact unconditional confidence limits that guarantee the level of coverage probability (calculated using StatExact). But, for instance, the method of Santner and Snell (SS) was used in a previous version of StatExact and is available in SAS version 9.2 [25]. More details of those methods can be found in the corresponding articles.

## Results

Eleven noninferiority trials were selected from criteria described above and table 1 summarizes their main characteristics [6], [9], [10], [11], [12], [13], [26], [27], [28], [29]. EASIER results were published in 2009 but the study was included because both it involved a small sample size and provided a treatment difference very closed to 0. Such a situation may potentially provide quit different confidence intervals estimates. Studies are ordered by sample size from trials enrolling less than 100 patients per arm to 300 patients per arm. Primary endpoint was mainly achievement of an HIV-RNA <50 copies/mL measured at week 48 of follow-up although few studies used a slightly different endpoint.

**Table 1:** Descriptive information on 11 recent HIV noninferiority trials.

Studies	Patients	Comparison	Sample size	Hypothesis	Margin	Power	2-sided CI	Method
EASIER	trt-exp	RAL vs. ENF	85 vs. 84	p1 = p2 = 96%	10%	80%	95%	Farrington and Manning
KALESOLO	trt-exp	LPV/r alone vs. HAART	87 vs. 99	p1 = p2 = 90%	12%	80%	90%	Wald
NCT00162643	naive	EFV vs. LPV/r	95 vs. 94		12%		95%	Wald
PROGRESS	naive	RAL/LPV/r vs. TDF/FTC/LPV/r	101 vs. 105	p1 = p2 = 75%	20%	90%	95%	Exact CZ
MONOI	trt-exp	DRV/r alone vs. DRV/r-regimen	112 vs. 113	p1 = p2 = 90%	10%	80%	90%	Wald
MONET	trt-exp	DRV/r alone vs. DRV/r-regimen	123 vs 123	p1 = p2 = 90%	12%	80%	95%	Wald
SPIRAL	trt-exp	RAL vs. PI/r	139 vs. 134	p1 = p2 = 85%	12.5%	80%	95%	Newcombe
Switchmrk 1 and 2	trt-exp	RAL vs LPV/r	172 vs. 174 175 vs. 178	p1 = p2 = 87.5%	12%	90%	95%	Miettinen and Nurminen
ODIN	trt-exp	DRV/r qd vs. DRV/r bid	294 vs. 296	p1 = p2 = 70%	12%	90%	95%	Wald
M06-802	trt-exp	LPV/r qd vs. LPV/r bid	300 vs. 299	p1 = p2	12%	>80%	95%	Wald

Trt-exp: treatment experienced.

### Clinical and statistical hypotheses

Hypotheses of success rates and power were either found in original articles or provided by investigators after request. For one trial, however, information on success rates and power were missing. Hypotheses of success rates varied from 70% to 96% and should be consistent with data from previous studies using both similar treatment regimen and population of patients. In some cases, however, it is difficult to anticipate success or failure rates with a new combination therapy or with a current combination but in a new population of patients.

Most of the noninferiority margin was fixed at 12% or around 12% (two studies had a 10% margin and one a 12.5% margin). The PROGRESS study used an unconventional 20% margin to investigate the efficacy of a new combination (lopinavir/r+raltegravir) [9]. The power is one of the key points of a study and summarized by itself most of the statistical hypotheses. Despite a large noninferiority margin, the PROGRESS study has a 90% power. Then a margin of 12% in the PROGRESS study, with the same rates of success and sample size (n = 100/arm), would approximately lead to a low power of 50%.

Another key point is the type I error (α significance level) or equivalently the level of the confidence interval (CI). A 1-sided α = 0.025 corresponds to a 2-sided 95% CI. MONOI and KALESOLO studies used a 2-sided 90%. There is a wide use of a 2-sided 95%CI although a 2-sided 90% CI is deemed acceptable for the noninferiority hypothesis test [15]. In the two studies using a 2-sided 90%CI, a monotherapy with a ritonavir-boosted protease inhibitor was compared with a triple-drug regimen [12], [28]. In this comparison, it is obvious that the efficacy of a single drug cannot be better than a triple-drug regimen justifying the use of a 1-sided α = 0.05. Importantly, MONET and MONOI had equivalent power because in MONOI study the use of a smaller 2-sided CI (90%) is balanced by a smaller noninferiority margin (10%) compared with MONET that used a 95% 2-sided CI but with a 12% margin [11], [12].

### Population sets and analysis

The MONET study excluded from the PP analysis mainly patients on the basis of violation of inclusion criteria, while the MONOI study excluded patients on the basis of major protocol violations, including violation of inclusion criteria and violation of the protocol post randomization [11], [12]. For instance, in the MONOI study while discontinuation was a cause of failure in the definition of the primary endpoint, patients who discontinued study treatment without virologic failure or severe adverse event were excluded from the PP population [12]. The KALESOLO study used a ‘switch included’ sensitivity analysis where all patients who intensified their antiretroviral treatment in the monotherapy arm were considered as success if they had an HIV-1 RNA <50 copies/ml at week 48 [28]. All other studies used an ‘on-treatment’ analysis considering only patients still receiving the assign treatment or an observed analysis which is quit similar although few distinctions can be found in the way missing data were handled.

**Figure 1:**
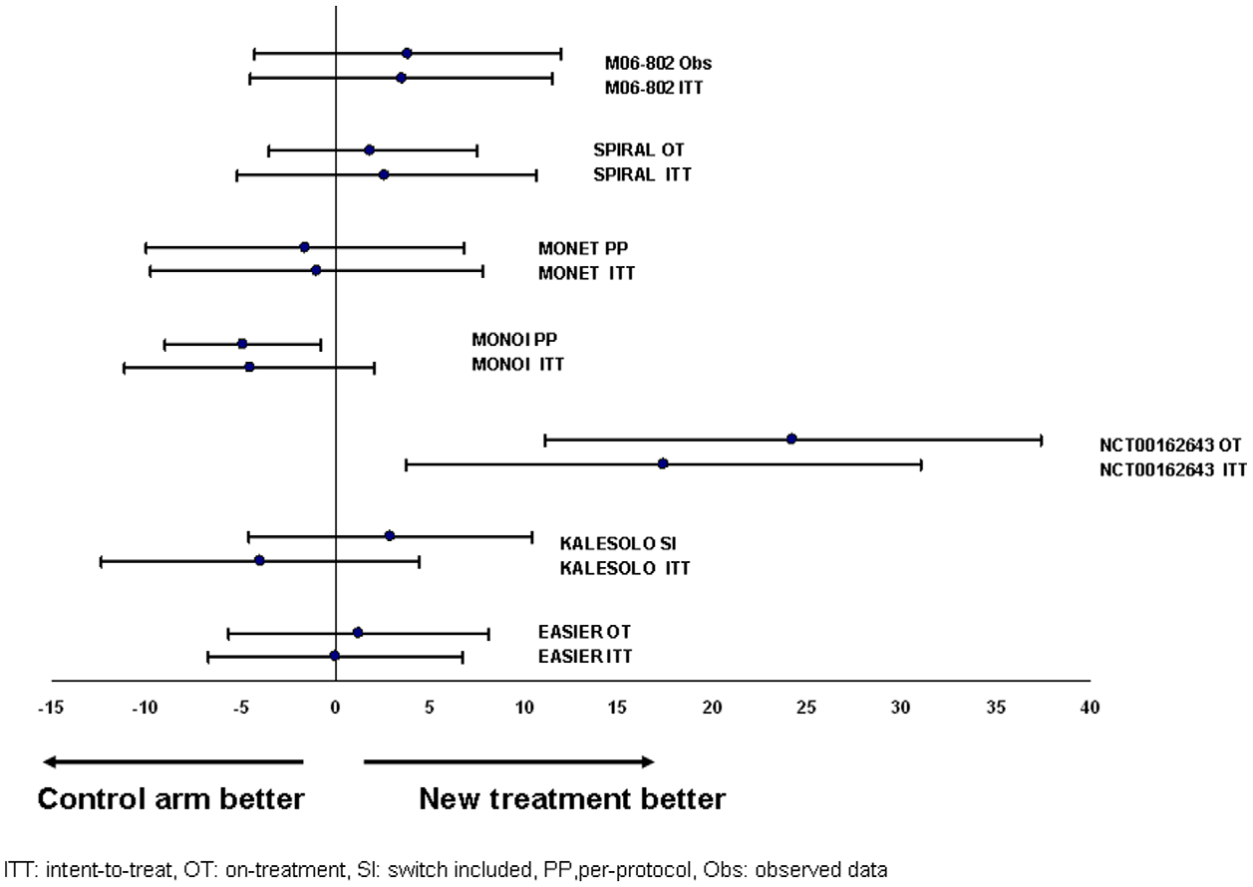
Difference in success rates with confidence intervals in 9 trials presenting both an ITT and a non-ITT analyses.

Results of the seven trials considering a non-ITT analysis in addition to the ITT analysis are displayed in Figure 1. Most of the trials provided very similar results between ITT and non-ITT analyses. KALESOLO exhibits an important difference between the ITT and the switch-included analysis but the latter analysis is special and had received criticisms [30]. One can be somewhat puzzled by results of the NCT00162643 study for a noninferiority design that exhibit a strong benefit of the new treatment group like a superiority trial.

### Confidence intervals estimates

The four methods, briefly described above, were then applied to data of the 11 trials (tables 2 and 3). As discussed above results based on the MN method were very similar to those provided by the FM method and are not displayed. Original results published or presented are indicated in bold. As expected, the four methods provided more different confidence intervals with 100 patients randomized per arm rather than with 300 patients per arm. In the EASIER study, the Wald method estimated the smaller confidence interval while the FM method provided the larger one. Then, although the conclusion of this study is not affected by the choice of the method, those methods led to very distinct confidence intervals. The sample size, however, is not the only factor that influenced confidence interval estimates. There is more discrepancy between the confidence interval estimates in the MONOI study than in the NCT00162643 study. The four methods provided almost similar confidence intervals in studies involving more than 200 patients per treatment arm.

**Table 2:** Results of 6 HIV noninferiority trials.

					Confidence intervals			
Studies	Analysis	Margin	Results	δ	Wald	Exact CZ	Newcombe	FM
EASIER	ITT	−10%	98.8% vs. 98.8%	0.01%	−3.3% to 3.3%	−5.6% to 5.7%	−5.3% to 5.4%	**−6.7% to 6.8%**
EASIER	OT	−10%	98.8% vs.100%	−1.22%	−3.6% to 1.2%	−7.3% to 3.4%	−6.6% to 3.4%	**−8.1% to 5.6**
KALESOLO	ITT	−12%	83.9% vs. 87.9%	−3.97%	***−12.4% to 4.5%***	*−14.7% to 6.3%*	*−12.7% to 4.5%*	*−12.7% to 4.8%*
KALESOLO	Switch included	−12%	90.8% vs. 87.9%	2.93%	**−4,5% to 10.4%**	−7.0% to 12.3%	−4.8% to 10.5%	−5.4% to 11.3%
NCT00162643	ITT	−12%	70.5% vs. 53.2%	17.33%	**3.7% to 31%**	3.3% to 30.7%	3.5% to 30.3%	3.5% to 31.1%
NCT00162643	OT	−12%	85.9% vs. 61.7%	24.2%	**11.1% to 37.3%**	10.4% to 37.1%	10.6% to 36.6%	10.2% to 38.1%
PROGRESS	ITT	−20%	83.2% vs. 84.8%	−1.6%	−11.6% to 8.4%	**−12.0% to 8.8%**	−11.8% to 8.5%	−12.2% to 9.0%
MONOI	ITT	−10%	87.5% vs. 92.0%	−4.54%	***−11.2% to 2.1%***	*−13.0% to 4.1%*	*−11.4% to 2.2%*	*−11.5% to 2.5%*
MONOI	PP	−10%	94.1% vs. 99.0%	−4.9%	−9.1% to −0.8%	*−11.4% to 0.2%*	*−10.1% to −0.6%*	*−10.1% to 0.3%*
MONET	ITT	−12%	84.3% vs. 85.3%	−1.0%	**−9.8% to 7.8%**	−10.1% to 8.3%	−9.9% to 7.9%	−10.1% to 8.1%
MONET	PP	−12%	86.2% vs. 87.8%	−1.6%	**−10.0% to 6.8%**	−10.4% to 7.4%	−10.2% to 6.9%	−10.4% to 7.1%

ITT: intent-to-treat, PP, per-protocol, OT on-treatment.

Original results are bolded and values in italic indicate inconclusive results (noninferiority not demonstrated).

**Table 3:** Results of 5 HIV noninferiority trials.

					Confidence intervals			
Studies	Analysis	Margin	Results	δ	Wald	Exact CZ	Newcombe	FM
SPIRAL	ITT	−12.5%	89.2% vs. 86.6%	2.6%	−5.1% to 10.4%	−5.5% to 10.9%	**−5.2% to 10.6%**	−5.6% to 10.9%
SPIRAL	OT	−12.5%	96.9% vs. 95.1%	1.8%	−3.9% to. 7.5%	−3.7% to 7.6%	**−3.5% to 7.5%**	−5.0% to 8.6%
Switchmrk 1	ITT	−12%	80.8% vs. 87.4%	−6.54%	*−14.2% to 1.14%*	*−14.4% to 1.3%*	*−14.3% to 1.2%*	***−14.3% to 1.2%***
Switchmrk 2	ITT	−12%	88.0% vs. 93.8%	−5.82%	−11.8% to 0.15%	*−12.2% to 0.3%*	*−12.1% to 0.3%*	***−12.2% to 0.5%***
ODIN	ITT	−12%	72.1% vs. 70.9%	1.2%	**−6.1% to 8.5%**	−6.2% to 8.6%	−6.1% to 8.4%	−6.1% to 8.5%
M06-802	ITT	−12%	55.3% vs. 51.8%	3.5%	**−4.5% to 11.5%**	−4.6% to 11.6%	−4.5% to 11.4%	−4.4% to 11.4%
M06-802	Observed data	−12%	76.0% vs. 72.2%	3.8%	**−4.3% to 11.9%**	−4.4% to 12.1%	−4.3% to 11.9%	−4.3% to 12.0%

ITT: intent-to-treat, PP, per-protocol, OT on-treatment.

Original results are bolded and values in italic indicate inconclusive results (noninferiority not demonstrated).

### Conclusion of the trials

Values in italic in Tables 2 and 3 indicate the method leading to inconclusive results, i.e., the noninferiority could not be demonstrated. Fortunately, a complete agreement of conclusions was mainly observed with the use of the four distinct methods except in two situations. In the MONOI study, the PP analysis using the Wald method, as planned in the protocol, demonstrated the noninferiority of the darunavir/ritonavir monotherapy to darunavir/r triple therapy but the three other methods were inconclusive. Similarly, in the Switchmrk 2 study the noninferiority of a raltegravir-based regimen to a lopinavir/ritonavir-based regimen was demonstrated with the Wald method but not with the three other methods.

### Widths of confidence intervals of ITT versus PP: an artifical example

The reason of a larger confidence interval for the ITT analysis compared with the PP analysis is given in Table 4. Table 4 illustrates how sample sizes and level of success rates, for a fixed treatment difference, impact the width of the confidence interval. We computed the width of the confidence interval with the Wald method in different situations where δ = −5%. The width of the confidence interval is strongly affected by the levels of success rates in the two groups. For example, it changes from 0.103 when p_1_ = 90% vs. p_2_ = 95% to 0.196 when p_1_ = 50% vs. p_2_ = 55% (Table 4). Potential difference between ITT and PP analyses can be illustrated with the following hypothetical trial. Consider a trial with δ = −5% in both ITT and PP analyses but with a success rate of 85% and 90% in the control group in the ITT and PP population, respectively. With n_1_ = n_2_ = 200, the width of the confidence interval is 0.149, with n_1_ = n_2_ = 190 in the PP analysis, corresponding to a 5% loss of patients, the width of the CI is 0.133, increasing then probability of demonstrating the noninferiority with a similar treatment difference (Table 4). Similar trends were found with the three other statistical methods.

**Table 4:** Computation of the width of 95% confidence interval according to different sample size and success rates for a fixed treatment difference of −5%.

		Width of 95% confidence interval for δ = −5%					
		ITT	PP				
		N1 = N2	N1 = N2				
p2	p1	200	190	180	170	160	150
**0.95**	**0.90**	0.103	0.105	0.108	0.111	0.115	0.119
**0.90**	**0.85**	0.129	0.133	0.136	0.140	0.145	0.149
**0.85**	**0.80**	0.149	0.152	0.157	0.161	0.166	0.172
**0.80**	**0.75**	0.163	0.168	0.172	0.177	0.183	0.189
**0.75**	**0.70**	0.175	0.179	0.184	0.190	0.195	0.202
**0.70**	**0.65**	0.183	0.188	0.193	0.199	0.205	0.212
**0.65**	**0.60**	0.190	0.194	0.200	0.206	0.212	0.219
**0.60**	**0.55**	0.194	0.199	0.204	0.210	0.216	0.223
**0.55**	**0.50**	0.196	0.201	0.206	0.212	0.219	0.226

		N1 = N2	N1 = N2				
p2	p1	100	95	90	85	80	75
**0.95**	**0.90**	0.145	0.149	0.153	0.158	0.163	0.168
**0.90**	**0.85**	0.183	0.188	0.193	0.198	0.204	0.211
**0.85**	**0.80**	0.210	0.216	0.222	0.228	0.235	0.243
**0.80**	**0.75**	0.231	0.237	0.244	0.251	0.258	0.267
**0.75**	**0.70**	0.247	0.254	0.261	0.268	0.276	0.285
**0.70**	**0.65**	0.259	0.266	0.273	0.281	0.290	0.299
**0.65**	**0.60**	0.268	0.275	0.283	0.291	0.300	0.309
**0.60**	**0.55**	0.274	0.281	0.289	0.297	0.306	0.316
**0.55**	**0.50**	0.276	0.284	0.291	0.300	0.309	0.319

ITT: intent-to-treat, PP, per-protocol.

### Widths of confidence intervals of the 11 trials

In general, the Wald method is known as being conservative, i.e., producing smaller width of confidence intervals compared with other methods. Table 5 demonstrated, however, than the Wald method did not estimates systematically shortest confidence intervals. Considering the 18 ITT and non-ITT analyses the Wald method provided the shortest confidence interval in 13 analyses, the Newcombe method in 4 analyses, and the FM in one. The Newcombe method estimated shortest confidence intervals when δ was very large (δ>17%). Largest confidence intervals were estimated by the FM method in 8 analyses and by the Exact (CZ) method in 10 analyses.

**Table 5:** Width of confidence intervals for the 11 HIV noninferiority trials according to the different statistical methods.

	Width of confidence intervals							
	ITT				non-ITT			
Studies	Wald	Exact CZ	Newcombe	FM	Wald	Exact CZ	Newcombe	FM
EASIER	0.065	0.112	0.106	**0.135**	0,048	0,107	0,100	**0,137**
KALESOLO	0.169	**0.210**	0.171	0.175	0,149	**0,193**	0,153	0,167
NCT00162643	0.273	0.274	0.268	**0.276**	0.262	0.268	0.261	**0.279**
PROGRESS	0.201	0.208	0.203	**0.212**				
MONOI ITT	0.132	**0.171**	0.136	0.137	0.098	**0.116**	0.106	0.104
MONET ITT	0.176	**0.184**	0.178	0.181	0.168	**0.178**	0.172	0.175
SPIRAL	0.155	0.163	0.158	**0.165**	0,098	0,113	0,111	**0,136**
Switchmrk 1	0.154	**0.157**	0.155	0.155				
Switchmrk 2	0.119	0.125	0.127	**0.127**				
ODIN	0.146	**0.147**	0.145	0.146				
M06-802	0.160	**0.161**	0.159	0.159	0,162	**0,165**	0,162	0,163

ITT: intent-to-treat.

Smaller value is underlined and larger value is bolded.

### Exact methods

Table 6 illustrates the difference in ‘exact’ confidence interval between the CZ and the SS methods. EASIER provided the most extreme situation since the confidence interval changes from [−5.6; 5.7%] with the CZ method to [−15.0%; 15.0%] with the SS method. Thus, the use of the SS method would lead to inconclusive results in the EASIER study. Results would also be inconclusive for the MONET and Swithmrk 2 studies. Large differences between those two methods were also found with trials including more than 100 patients per arm (SPIRAL). The exact SS method provided the largest confidence intervals in all situations explored in Tables 2 and 5.

**Table 6:** Comparison between two exact methods (CZ, Chang and Zhang and SS, Santner and Snell) applied to data from the 11 HIV noninferiority trials.

Studies	Analysis	Exact CZ	Exact SS	Studies	Analysis	Exact CZ	Exact SS
**EASIER**	ITT	−5.6% to 5.7%	*−15.0% to 15.0%*	**SPIRAL**	ITT	−5.5% to 10.9%	−9.3% to 14.6%
**EASIER**	OT	−7.3% to 3.4%	*−14.6% to 17.0%*	**SPIRAL**	OT	−3.7% to 7.6%	−10.6% to 14.3%
**KALESOLO**	ITT	*−14.7% to 6.3%*	*−18.3% to 10.5%*				
**KALESOLO**	Switch included	−7.0% to 12.3%	−11.5% to 17.2%				
**NCT00162643**	ITT	3.3% to 30.7%	2.7% to 30.8%	**Switchmrk 1**	ITT	*−14.4% to 1.3%*	*−17.1% to3.9%*
**NCT00162643**	OT	10.4% to 37.1%	8.4% to 38.6%	**Switchmrk 2**	ITT	*−12.2% to 0.3%*	*−16.1% to 4.8%*
**PROGRESS**	ITT	−12.0% to 8.8%	−15.1% to 12.2%	**ODIN**	ITT	−6.2% to 8.6%	−6.9% to 9.2%
**MONOI**	ITT	*−13.0% to 4.1%*	*−17.4% to 8.6%*	**M06-802**	ITT	−4.6% to 11.6%	−4.7% to 11.5%
**MONOI**	PP	*−11.4% to 0.2%*	*−18.9% to 9.3%*	**M06-802**	Observed data	−4.4% to 12.1%	−5.5% to 13.0%
**MONET**	ITT	−10.1% to 8.3%	*−13.4% to 11.1%*				
**MONET**	PP	−10.4% to 7.4%	*−14.4% to 11.2%*				

ITT: intent-to-treat, PP, per-protocol, OT on-treatment.

Values in italic indicate inconclusive results (noninferiority not demonstrated).

## Discussion

This work investigated the impact of the statistical methods used in the analysis of HIV noninferiority trials. An optimistic view may consider that, from the 18 datasets (trial/set of population) analyzed by 4 different statistical methods, different conclusion of the results were draw in only 2 occasions. One remark, however, than in some datasets the different methods assessed very distinct confidence intervals. Conclusions were not altered by those different confidence intervals due to the point estimate of the treatment difference. It is obvious that an observed treatment difference far from the noninferiority margin will generally lead to demonstrate noninferiority whatever the method used. In the two datasets with discordant conclusions, the observed treatment differences were −4.9% and −5.82% corresponding to the midpoint between 0 and the noninferiority margin chosen.

The MONOI study provides an interesting situation since the PP analysis concluded to the noninferiority while the ITT was inconclusive. As discussed above, it is often admitted that the ITT analysis tends to dilute the treatment difference and then may lead to erroneously conclude of noninferiority for a drug that is truly inferior to the active control groups among compliers [15]. A general idea is also that the width of the confidence interval of the treatment difference for the PP analysis is larger than the ITT analysis, due to smallest sample sizes. Although it has be noted that low success rates observed in the ITT analysis are associated with larger variances and then to larger confidence intervals [18]. In the MONOI study, it is difficult to consider a dilution of the treatment effect since the two analyses provide very concordant results (−4.5% vs. −4.9%). Nevertheless, the ITT analysis failed to demonstrate noninferiority, whereas the PP analysis showed noninferiority.

The regulatory agencies provide guidelines covering the statistical principles for clinical trials [16] including the choice of the noninferiority margin [31] and the points to consider on switching between superiority and non-inferiority [32]. The approach based on confidence intervals for difference in proportions is accepted but no specific statistical methods are recommended. It is expected that the full analysis set and the per protocol set lead to the same conclusions to increase confidence in the trial results [16]. In the MONOI study, however, treatment differences estimates in the ITT and PP anlyses were almost similar whereas leading to difference conclusions. Superiority trials may not serve to demonstrate non-inferiority and the main conclusion of non-inferiority trials should be stated whether the non-inferiority is demonstrated or not. A recent HIV equivalence trial is confusing since for the two pairwise comparisons the two upper limits of the 95% CI were greater than the prespecified margin whereas the authors concluded that the two regimens had ‘similar’ antiviral activity [33].

The choice of the noninferiority margin is a key point and should be based upon a combination of statistical reasoning and statistical judgement [31]. The link with statistical hypotheses was best illustrated with the PROGRESS study that provides a similar power than the ODIN study with a much larger margin (20% vs. 12%). In general, it is admitted that the margin should be smaller than the clinically relevant effect [15], [34]. The margin should also be linked with the severity of the primary endpoint. In the HIV trials, mortality and clinical endpoint are rarely used since 1997 and the consequence of virologic/treatment failure as primary endpoint in current HIV trials is a treatment modification. In most cases, patients who changed all or one compound of their regimen are subsequently in therapeutic success with HIV-1 RNA <50 copies/mL [11], [12]. One can suspect than a margin lower than 10% would be used with a primary endpoint based on mortality or occurrence of serious clinical events. Noninferiority trials accept that a new treatment should be worse than the standard by an amount less than the prespecified margin on the premise that it has some other advantage (lower toxicity, greater ease of administration, better adherence, reduced cost). A consequence is that, for a given power, a larger margin should be associated with some larger advantages.

Comparison between the two ‘exact’ methods is confusing. First the difference between these two methods is more important than between any exact and any non-exact method. Second, the term ‘exact’ may be very confusing for clinicians who consider that an ‘exact’ method is definitive and that no improvement can be made. In general, one considers that exact methods are better or more appropriate than non-exact methods. But which exact method should be used? Chan and Zhang suggested their method because they pointed out that the SS method was overly conservative [21]. Few illustrative examples and a simulation study in a limited number of situations, both based on small sample size (n≤20), showed an improvement of the CZ method over the SS method [21]. Our results show that even with larger sample size, confidence intervals based on the SS are very conservative suggesting the use of the exact CZ method.

Interestingly some authors have suggested that approximate is better than exact for interval estimation of binomial proportions [35], [36]. So again, which method should be used? A first work compared three methods (Wald, Dunnett and Gent, FM) for testing therapeutic equivalence in a clinical setting (n>>20) [37]. The authors concluded that both Wald and FM methods can be used for Δ_L_<p_2_/2. For quite unusual configurations, the Wald method performed even better [37]. Newcombe provided the largest investigation of methods for interval estimation for the difference between two proportions [38]. Eleven methods were compared in a very large setting covering a wide range of parameters (p_1_,p_2_) but mainly with low sample size (n = 5 to 50). He concluded that the Newcombe method achieved better coverage probability than any simple methods. Nevertheless, none of the exact method was included in the comparison. In a last work, Barker and colleagues compared 8 methods for testing equivalence in the case of difference of two binomial proportions, including the Wald and Newcombe methods but not the FM and CZ or SS exact methods [39]. Surprisingly, the conclusion of their simulation study did not accurately reflect results shown in their tables. For example, they concluded that when n_1_ = n_2_ = 50 the WALD method is not anti-conservative this is true because this approach is very conservative (cf reference [39], pp281, Table 2 n = 50). Those different works highlighted the difficulty to choose a method although the exact CZ, Newcombe and FM methods seem the most appropriate.

A limitation of the study is that we did not applied all the statistical methods that have been proposed to estimate confidence intervals for the difference between independent proportions. The four methods, however, where the methods used in HIV noninferiority trials publisehed in 2010 and represent a large panel of methods. It can also be argued that each method used for the analysis was also used for sample size/power determination. And then only the planned method should be used as corresponding to a given sample size and power. In fact, the four methods provide almost similar sample sizes. For example, with p_1_ = p_2_ = 0.90, α = 0.025 (one-sided) 1-β = 90%, and Δ_L_ = 0.10, the sample size per group is 189, 204, 200 and 201 with the Wald, FM, Newcombe and Exact CZ, respectively, and 441, 441, 445, and 447, respectively with p_1_ = p_2_ = 0.70 (see also reference [22]). Of note sample size for the Newcombe method is obtained by simulation [NQueryAdvisor].

In conclusion, the choice of the statistical methods may lead to different confidence intervals estimates, especially in trials with low or moderate samples size. The exact CZ, Newcombe and FM methods seem the most appropriate methods although further investigation comparing at least those three methods in a clinical trials setting will be helpful to determine the best method according to different scenario. Choice of the methods has low or no impact on determination of the sample size.
